# Retrospective Studies with Inconsistent Data: Results, Conclusions and Recommendations should be taken with a Grain of Salt

**DOI:** 10.4103/1319-3767.77235

**Published:** 2011

**Authors:** Ibrahim AlTraif

**Affiliations:** Department of Hepatobiliary Sciences, Sciences and Liver Transplantation, King Abdulaziz Medical City, Riyadh, Saudi Arabia

Gastroesophageal varices are present in about 50% of cirrhotic patients, with a 40% prevalence rate in Child Pugh (CP) class A cirrhotic patients and 85% among CP class C patients. Variceal hemorrhage occurs at a yearly rate of 5-15%, and the most important predictor for bleeding is the size of the varices, the presence of red wales, and decompensated cirrhosis.[1] The management of variceal hemorrhage has greatly improved in the past two decades, with mortality rates reducing from 40% in earlier studies to 10-15% in more recent studies.[1] This is due to an improvement in the general management of these patients and the utility of endoscopic band ligation (EBL) to arrest bleeding. EBL has replaced sclerotherapy which was associated with high rebleeding and complications rates, and required more endoscopic sessions for variceal eradication.[1] The improvement in outcome is also attributed to the advances made in liver transplantation thereby serving to salvage patients and prevent future rebleeding episodes, particularly in advanced CP class C cirrhotic patients. Finally, prophylactic antibiotic usage, including intravenous erythromycin prior to endoscopy for better mucosal visualization,[2] advances in endoscopic techniques, and the introduction of trans-jugular porto-systemic shunts (TIPS) have also contributed to the overall improved outcome.

Currently, the recommendations regarding primary prophylaxis for variceal bleeding are illustrated by Thomas *et al*, who reported in a meta-analysis of endoscopic variceal ligation for primary prophylaxis of esophageal variceal bleeding compared with untreated controls that prophylactic ligation reduces the risks of variceal bleeding and mortality.[3] Compared with β-blockers, ligation reduces the risk for first variceal bleed but has no effect on mortality. Based on this, prophylactic ligation should be considered for patients with large esophageal varices (high risk for bleeding) who cannot tolerate β-blockers.[1]

Whether prophylaxis with EBL in addition to β-blockers would be more beneficial has not been established, in line with the recent work of Lo *et al*, who showed in a randomized trial of cirrhotic patients with high-risk esophageal varices but without bleeding history that band ligation plus nadolol compared to nadolol alone was not superior and may be associated with more complications.[4] With seventy patients in each group, and a median follow-up duration of 26 months, esophageal variceal bleeding occurred in 10 patients (14%) in the combined group and in 9 patients (13%) in the nadolol group. Adverse events were noted in 48 patients (68%) in the combined group and 28 patients (40%) in the nadolol group. Sixteen patients in each group died, mostly from hepatic failure. This study served to highlight that there is no evidence to support combined EBL plus β-blockers for primary variceal prophylaxis.

In relation to secondary prophylaxis, Gonzales et al, in a meta-analysis of 18 well-designed studies including 1125 patients, reported 19% rebleeding rate with combination of drugs and EBL while it was 28% with EBL alone.[5] This difference in outcome was not statistically significant.

In this issue of the Journal, Ouakaa-Kchaou and colleagues report a retrospective study of EBL in the prevention of variceal bleeding in 603 Tunisian patients.[6] In this multicenter trial conducted over 10 years, the authors included 49 patients (8% of studied subjects) undergoing EBL as primary prophylaxis and 554 patients (constituting the remaining 92% of patients) undergoing EBL as secondary prophylaxis, in which 126 (21%) patients had recurrent bleeding. Patients also received propranolol simultaneously as secondary pharmacologic prophylaxis.

Essentially, in order to evaluate a study, its strengths and weaknesses must be analyzed critically to determine whether its results, conclusion, and recommendation should be taken at face value. While the authors included a large number of patients, the study suffers from several weaknesses. Primarily, it includes patients undergoing EBL for primary as well as secondary prophylaxis of variceal bleeding. These are two obviously different groups of patients with a vastly different disease course and outcome and as such should not be studied concomitantly.

Secondly, this is a multicenter study saddled with the inherent difficulties of its retrospective nature including patient selection bias, data collection problems, adverse events underreporting, follow-up data inconsistency, and major problems in measurement of outcome. More specifically, the study is burdened with a lack of uniform treatment strategy among centers, resulting in inter-center variability (differences between treating centers) and intra-center variability (differences among care-givers of the same center). Since the study reports a follow-up of 212 months (17.7 years) in addition to the 4 years since the study was completed, it is feasible that some of the patients may have undergone endoscopic sclerotherapy, at least initially, or received single band ligations with overtube, while others received multiple speed band sessions and other forms of endoscopic combination therapy. Intriguingly, the authors report a follow-up of 212 months and yet they contradict themselves by stating that the study was conducted from 1998 to 2007, essentially a 10-year duration. While following a mental algorithm of the study inclusions, it becomes increasingly difficult to follow the patient flow and their outcomes at different time points in the study, thus making it impossible to analyze the results in a meaningful manner.

Thirdly, the institution of β-blocker therapy was not consistent; we cannot ascertain if some patients received it as primary prophylaxis and/or only as secondary prophylaxis. Moreover, the rate of discontinuation, adverse event reporting and achievement of therapeutic dosing has not been mentioned, as this may impact on overall bleeding rates.[1,7] Future research in this area should focus on a comparison of band ligation with β-blockers to determine the effect on mortality and ascertain the cost-effectiveness of ligation.

The study by Ouakaa-Kchaou *et al*[6] has several problems at different levels including, but not limited to, the study design are generally not retrospective in nature, have simple specific research questions to be answered, and not several questions being addressed, with the eventual likelihood that none of them would be answered adequately. Ouakaa-Kchaou *et al*, have been unable to convincingly demonstrate that endoscopic band ligation is beneficial in either primary or secondary prophylaxis of esophageal variceal bleeding. The study is a case in point that skewed data arising from arising from non-robust reporting can only be taken with a grain of salt. Generally, medical journals should take the view of not publishing retrospective studies unless reporting an issue of significant clinical relevance which in itself cannot be undertaken in a prospective manner or doing so would be unethical or prohibitively expensive; or the findings concern a rare condition or encompasses the findings of a large database.
